# Image super-resolution reconstruction algorithm based on multi-scale recursive attention and feature fusion

**DOI:** 10.1371/journal.pone.0333398

**Published:** 2025-10-07

**Authors:** Haixia Liu, Mingliang Wang

**Affiliations:** 1 Keyi College, Zhejiang Sci-Tech University, Shaoxing, China; 2 Zhejiang Silk Technology Co., Ltd., Hangzhou, China; 3 College of Materials and Textiles, Zhejiang Sci-Tech University, Hangzhou, China; Islamia University of Bahawalpur: The Islamia University of Bahawalpur Pakistan, PAKISTAN

## Abstract

Image super-resolution reconstruction is one of the important application branches of computer vision in many fields. However, in actual complex environments, images are often subject to various interferences, leading to severe distortion in the reconstructed images. To address this issue, this study innovatively combines multi-scale feature extraction (MSFE) and attention feature fusion (AFF). After optimization, the multi-scale recursive attentional feature fusion block (MSRAFFB) and MSRAFFB Network (MSRAFFB-Net) application algorithms are proposed. Simulation experiments on standard datasets demonstrated that MSRAFFB was crucial for enhancing the overall performance of the algorithm, improving reconstruction quality over the baseline. Additionally, through reasonable network structure design (such as increasing module depth and branch complexity), MSRAFFB-Net effectively reduced reconstruction error and improved perceptual quality. The algorithm exhibited high reconstruction accuracy across different magnification factors. Furthermore, to assess the algorithm’s performance in more realistic and complex environments, the study conducted performance experiments in actual application scenarios. The results indicate that the algorithm can effectively improve the resolution and visual quality of reconstructed images while preserving the original image’s characteristic information. In summary, the proposed algorithm significantly enhances the accuracy and robustness of image reconstruction, holding positive implications for advancing the application of computer vision technology in various real-world scenarios requiring high-resolution image processing.

## 1. Introduction

The advancement of image digitization has led to unprecedented improvements in image processing, analysis, and reconstruction techniques [1]. Image reconstruction can enhance the details of the original image and accelerate the processing speed of image information, which is well utilized in medical imaging and remote sensing detection, and is also a research focus in computer vision (CV) [2]. Image super-resolution reconstruction (ISRR) is an important deepening application direction of image reconstruction technology. According to the difference in the number of input images, it can be segmented into single-image super-resolution (ISR) and multiple ISR [3]. The latter is widely used due to its lower requirements for image processing and cost savings [4–5]. However, with the increasing complexity of application scenarios, traditional ISRR algorithms are unable to meet practical needs in real motion blur scenarios [6] and low-light noise variations [7]. For example, the image reconstructed by the nearest neighbor interpolation algorithm has rough edges and details, which can easily lead to block effects [8]. The iterative backprojection algorithm has slow convergence speed, long computation time, and is prone to getting stuck in local optima [9]. The bicubic interpolation, though widely used, exhibits limited high-frequency feature representation capability, resulting in blurring and ringing interferences [10]. These issues seriously affect the efficiency and accuracy of ISRR. Therefore, this study starts with the effective extraction and processing optimization of original image features, focusing on the basic logic and excellent performance of modules such as multi-scale feature extraction (MSFE), and proposes the multi-scale recursive attention feature fusion block (MSRAFFB). The research addresses three key challenges in ISR: insufficient MSFE in traditional algorithms leading to edge distortion and detail loss [11], degraded reconstruction quality and high computational complexity of existing methods under complex noise conditions [12], and practical limitations due to large-scale deep network parameters and inefficient training [13]. The research objective is to construct an integrated algorithm of multi-scale recursive attention and feature fusion to improve the accuracy of complex scene reconstruction. This algorithm can improve noise robustness by using attention-based hierarchical feature fusion to enhance resistance to noise interference and detail preservation. In addition, the performance advantages and generalization ability of the algorithm can be verified through peak signal-to-noise ratio (PSNR) and structural similarity index (SSIM) metrics and practical applications. Among them, PSNR measures the quality of signal reconstruction by calculating the mean square error between the original signal (e.g., image, audio) and the reconstructed signal. SSIM is used to measure the similarity between two images in terms of brightness, contrast, and structural information. The unique contribution of this research is the proposed MSRAFFB module, which integrates MSFE and attention feature fusion (AFF) to enhance detail capture and feature representation through recursive structures. MSRAFFB-Net, built upon the MSRAFFB network, effectively suppresses noise interference, improves reconstruction accuracy, and demonstrates superior noise robustness and reconstruction accuracy in real-world scenarios by preserving original image features and enhancing resolution.

The content has four sections. Section 1 introduces the current research on the logic and algorithms of ISRR worldwide. Starting from modules such as MSFE, AFF, and residual network (ResNet), section 2 establishes an accurate and efficient MSRAFFB-Net algorithm. Section 3 provides numerical examples and practical application analysis of the proposed reconstruction algorithm to verify its reliability. Section 4 provides a comprehensive summary and analysis of the article.

## 2. Related works

CV technology is advancing on a large scale in various industries, and the application of image reconstruction in fields such as automatic detection and intelligent driving is rapidly increasing [14]. ISRR is a key technology for improving image quality and an important application direction that needs to be continuously expanded and deepened in image reconstruction [15]. However, in practical work, ISRR performance exhibits significant fluctuations under varying noise levels and motion blur interferences [16], so many researchers are improving this issue [17]. In the domain of generative adversarial network (GAN)-based methods, in the generative model-driven approach, C. Saharia et al. put forth a method for achieving ISR through repeated refinement to address issues such as difficulty in removing noise in traditional image reconstruction algorithms. This method could achieve denoising process through random iteration [18]. H. Li et al. proposed a novel single-ISR diffusion probability model to address issues such as excessive smoothing and large model size in the process of image reconstruction. This model improved the richness of details in the output results while reducing the model size [19]. To address the shortcomings of existing ISR algorithms in extracting rich image features and preserving true high-frequency details, Zhu F et al. proposed an improved GAN algorithm that could restore image details more effectively and reduce distortion [20]. In these methods, researchers use adversarial training or diffusion processes to overcome the difficulties of noise reduction and oversmoothing in traditional methods through iterative optimization or probabilistic modeling, thereby enhancing details and reducing distortion and model complexity. To address the issue of insufficient utilization of complementary advantages between feature maps, channels, and pixels in transformer-based and attention mechanism enhancement methods, W. Zhang et al. proposed a cascaded visual attention network (CVANet) that effectively improved the efficiency of ISRR [21]. In response to the lack of constraints in the mapping process from low-resolution (LR) to HR, X. Liu proposed a self-attention negative feedback network to achieve ISRR of real-time images [22]. J. N. Su et al. proposed a globally learnable attention mechanism to address the phenomenon that low similarity non-local textures can provide more accurate and rich details, which improved the ISRR efficiency of images with different degradation types (such as blur and noise) [23]. In such methods, researchers mainly use Transformers to optimize feature interactions, improve feature complementarity, constrain low-high resolution mapping, and enhance the recovery efficiency of blurry/noisy images.

In multi-scale feature fusion methods, to cope with the significant increase in parameter counts caused by deep convolutional neural networks (CNNs), Y. Chen et al. proposed a single-ISR network based on 2-level nested residual blocks, which reduces the computational complexity of ISRR [24]. To address the problem that existing ISR methods were unsuitable for devices with limited computing power due to their numerous model parameters and high computational cost, X. Guo et al. proposed a lightweight multi-dimensional feature fusion network. This network effectively reduced model complexity and improved reconstruction performance [25]. In guided/multimodal fusion methods, R. Ran et al. proposed a universal fusion framework with high-resolution (HR) guidance to address the poor generalization performance of traditional image reconstruction algorithms. This method could significantly reduce network parameters while improving image reconstruction performance [26]. D. Cheng et al. proposed an image reconstruction method based on light-guided cross-fusion to address the issue of overexposure in ISRR under uneven lighting conditions, which improved the image reconstruction model’s resistance to interference from complex lighting conditions [27]. In the network structure optimization approach, C. Tian et al. proposed an enhanced super-resolution group to address the high dependence of traditional image reconstruction algorithms on deeper network architectures. This method was applicable to various image reconstruction models of different sizes [28]. To solve the problem of unrealistic texture in reconstructed images using traditional deep learning-based ISRR methods, L. Fu et al. introduced a method based on instance spatial feature modulation and a feedback mechanism. This method could achieve more realistic textures in reconstructed image instances [29]. In summary, researchers around the world have noted issues present in the operation of ISRR algorithms and have undertaken numerous research efforts to address these problems. To systematically compare the technical characteristics of emerging methods, the study summarizes representative work in recent years. The study analyzes the innovativeness and limitations of different methods through structured classification, which provides potential paths for performance benchmarking and cross-method optimization for research, as shown in Table 1.

**Table 1:** Recent advances in ISRR.

Method	Grouping	Advantages	Disadvantages	Key findings	References
ISR via iterative refinement (ISR-IR)	GAN-based	Uses iterative refinement steps and denoising score matching to generate high-quality super-resolution images	High computational complexity; yields diminishing returns on accuracy gains despite significant resource overhead	Performs well on multiple datasets with superior PSNR and SSIM metrics compared to traditional methods	[18]
CVANet	Transformer-based	Uses CVANets to effectively capture image details	More network layers and longer training time; creates latency barriers for real-time deployment despite perceptual quality benefits	Achieves good performance on multiple datasets, especially in detail recovery	[21]
Multi-level features fusion network (MFFN)	CNN-based	Enhances image quality through multi-level feature fusion	Relatively complex network structure; induces disproportionate computational burden relative to marginal quality improvements	Experimental results show superior visual and quantitative performance compared to some traditional methods	[24]
General CNN fusion framework via HR guidance (GuidedNet)	CNN-based	Proposes a general CNN fusion framework suitable for hyperspectral images	Utilization of spectral information in hyperspectral images is not fully optimized; entrenches high training costs without commensurate performance breakthroughs	Performs well in hyperspectral ISR tasks, effectively recovering spatial details	[26]
Enhanced group convolutional neural network (EGCNN)	CNN-based	Uses an EGCNN to improve computational efficiency	Requires high quality and quantity of training data; exhibits severe performance degradation under constrained data regimes	Achieves better PSNR and SSIM values on multiple standard datasets	[28]

While existing methods in Table 1 have improved image quality, they rarely optimize feature extraction and processing efficiency of original images. ISRR holds practical significance in fields like human-computer interaction by enhancing resolution for clearer detail display. This study integrates MSFE and AFF within a recursive network to propose the MSRAFFB-based ISRR algorithm, addressing accuracy and efficiency challenges in complex fuzzy image reconstruction through optimized feature utilization.

## 3. Methods and materials

This study first details the construction of the MSRAFFB module, then describes the application network built upon it, aiming to clearly reflect model’s logic and details. This section comprises two subsections: Subsection 1 proposes MSRAFFB by improving upon the analyzed MSFE and AFF modules; Subsection 2 combines MSRAFFB with recursive networks for secondary optimization of the image reconstruction algorithm, proposing MSRAFFB-Net to enhance feature processing performance.

### 3.1. Construction of msraffb module based on deep feature extraction optimization

**Fig 1:**
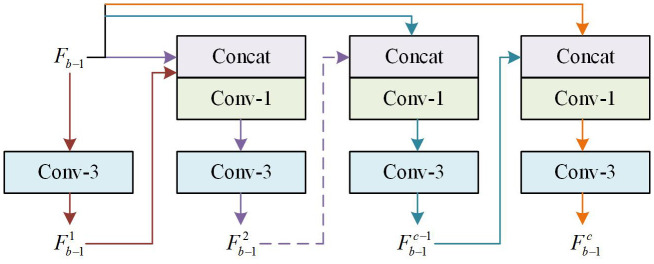
The structure of MSFE module.

Feature information extraction and processing are key operations for capturing image details and structures, and their efficiency determines the quality and clarity of reconstructed images [30]. However, traditional image reconstruction algorithms are often affected by multiple factors, resulting in low efficiency in extracting and utilizing feature information from the original image, leading to severe distortion of the output image [31]. In response to the above issues, this study proposes the MSRAFFB-Net algorithm. MSRAFFB is the core module of the algorithm, consisting of MSFE and AFF modules. Among them, MSFE extracts multi-scale features through multi-branch convolutions to enhance detail capture capabilities [32]. AFF uses squeeze-and-excitation (SE) attention to achieve adaptive channel weighting fusion [33]. The combination of the two balances computational efficiency and performance. Under the same task conditions, the combination of MSFE+AFF avoids the high complexity and memory overhead of traditional attention mechanisms and Transformer modules [34]. The MSFE structure is shown in Fig 1.

In Fig 1, the MSFE module consists of multiple branches. Each branch processes different scale information of input features. Each branch passes its output to the next branch, and also receives the output of the previous branch as input. The convolution kernel sizes for each branch of the MSFE module are set to 1*1 and 3*3, respectively, with the number of channels set to 64. This cross-branch connection forms a recursive feature refinement mechanism, in which 1 × 1 convolution is used for feature dimension reduction (reducing the amount of computation), while 3 × 3 convolution captures local spatial context information (enhancing detail perception). The choice of 1*1 and 3*3 convolution kernels and channels set to 64 in the MSFE module is based on their ability to effectively capture different scale features while maintaining computational efficiency [35]. The MSFE module can work effectively because it efficiently captures and integrates multi-scale information of input features through inter-branch connections, which enhances the ability of the network to represent features at different scales. In addition, its structure allows each branch to gradually accumulate and refine feature information during processing, further strengthening the network’s multi-scale perception ability and overall performance. The output of the MSFE module is shown in equation (1).


$$\left\{\begin{array}{l} F_{b-1}^{1}=f_{3*3}\left(F_{b-1}\right)\\ F_{b-1}^{2}=f_{3*3}\left(f_{1*1}\left(concat\left(F_{b-1,}F_{b-1}^{c}\right)\right)\right)\\ \vdots \\ F_{b-1}^{c-1}=f_{3*3}\left(f_{1*1}\left(concat\left(F_{b-1,}F_{b-1}^{c-2}\right)\right)\right)\\ F_{b-1}^{c}=f_{3*3}\left(f_{1*1}\left(concat\left(F_{b-1,}F_{b-1}^{c-1}\right)\right)\right) \end{array}\right)$$ (1)

**Fig 2:**
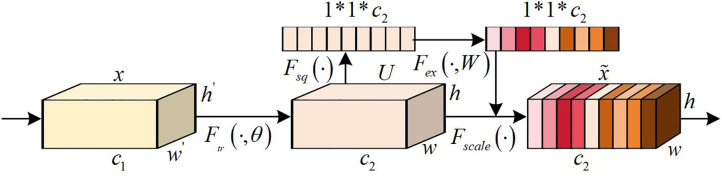
Structure of the SE attention mechanism.

In equation (1), $F_{b-1}^{c}$ is the c -th branch in the b -th MSRAFFB, c≥4. $F_{b-1}$ is the original input, and $F_{b-1}^{c-1}$ is the output of the (c − 1)-th branch. $f_{1*1}$ and $f_{3*3}$ are convolution kernels of sizes 1*1 and 3*3. However, the feature fusion method of the MSFE module is fixed, and its ability to capture local details in edge/texture areas is limited [36]. The AFF module adopts a channel space dual attention guided fusion strategy, which solves the problem of traditional weighted fusion ignoring semantic correlation between features. This module achieves a balance between detail enhancement and noise suppression through context aware dynamic weight allocation [37]. The SE attention mechanism is the core of the AFF module, as shown in Fig 2.

In Fig 2, if a feature map x with a height of $h^{\prime }$, a width of $w^{\prime }$, and a number of channels $c_{1}$ is input to the SE mechanism. The first step is to generate a new feature map U with a h, a w, and a $c_{2}$ through standard convolution operation $F_{tr}\left(\cdot ,\theta \right)$. Afterwards, U encodes the spatial features on the channel into global features through global average pooling and performs squeezing operations $F_{sq}\left(\cdot \right)$ and $h*w*c_{2}$ to convert them into $1*1*c_{2}$. The squeezing operation $F_{sq}\left(\cdot \right)$ is shown in equation (2).


$$x_{i}^{\prime }=F_{sq}\left(x_{i}\right)=\frac{\text{1}}{h*w}\sum_{i=1}^{h}\sum_{j=1}^{w}x_{i}\left(i,j\right)$$ (2)

In equation (2), $x_{i}^{\prime }$ represents the output of the squeezing operation. $x_{i}$ is the feature in the i -th channel. $x_{i}\left(i,j\right)$ is the value at position (i,j). The next step is to perform incentive operation $F_{ex}\left(\cdot ,W\right)$. It uses two fully connected neural networks to perform nonlinear transformations on the squeezed results to obtain weight values for different channels. The incentive operation $F_{ex}\left(\cdot ,W\right)$ is shown in equation (3).


$$y_{i}^{\prime }=F_{ex}\left(\cdot ,W\right)=R\left(w_{2}\sigma \left(w_{1}x_{i}^{\prime }\right)\right)$$ (3)

**Fig 3:**
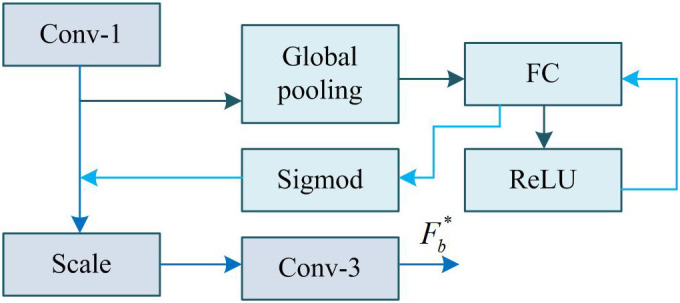
Framework of the AFF module.

In equation (3), $y_{i}^{\prime }$ is the output of the excitation operation. R is a rectified linear unit function (ReLU). $w_{1}$ and $w_{2}$ are weight coefficients learned in two fully connected layers (FCL). σ means the activation function Sigmod. Finally, through weighting operations, the output weights after excitation are multiplied with the original features channel by channel, and the previous features are rescaled $Y_{i}=y_{i}^{\prime }*x_{i}$ to gain a new feature map $\tilde{x}$. Among them, $Y_{i}$ is the output of SE. Therefore, SE obtains channel features through global average pooling, processes them through two FCLs and activation functions, and generates channel weights. This process essentially involves dynamically learning the importance weights of each channel: suppressing noise-dominated channels and reinforcing high-frequency detail channels (such as edges/textures). These weights are multiplied channel by channel with the original features to achieve reweighting of features, enhance important features, and suppress unimportant features [38]. This study applies SE in the AFF module, as shown in Fig 3.

**Fig 4:**
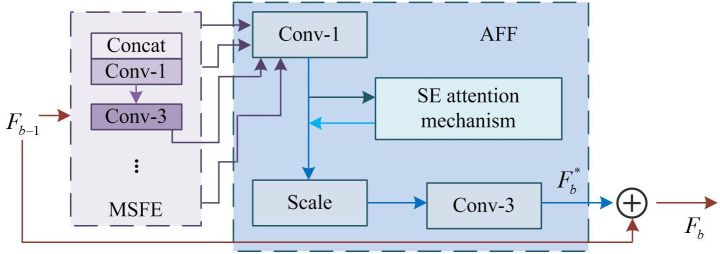
Structure of the MSRAFFB.

In Fig 3, the extracted features from all branches of MSFE will be used as inputs to the AFF module after dimensionality reduction through 1*1 convolution. Afterwards, the SE mechanism is used to fuse multi-scale feature information, learn the correlation between channels, adjust the weights of different channels, and thus increase the representation of useful features [39]. In Fig 3, an arrow pointing from ReLU to FCL indicates that the output of the ReLU activation function is used as an input to FCL, ensuring the flow and appropriate processing of data between different layers. Finally, the channels are restored utilizing a 3*3 convolution, and the resulting $F_{b}^{*}$ is used as the input for the next module. AFF works effectively because it uses the SE attention mechanism to obtain channel-wise features and to generate channel weights for reweighting the features. This approach highlights important features while suppressing noise and redundant information, thereby enhancing the discriminative power and robustness of the features. This study combines MSFE and AFF to propose MSRAFFB, whose structure is shown in Fig 4.

In Fig 4, input feature $F_{b-1}$ enters MSFE and undergoes convolution and concatenation operations on different branches to generate branch features such as $\left\{F_{b-1}^{1},F_{b-1}^{2},\ldots ,F_{b-1}^{c-1},F_{b-1}^{c}\right\}$. Afterwards, the branch features are input into the AFF module and processed by SE fusion to obtain the multi-scale feature fusion feature map $F_{b}^{*}$. $F_{b}^{*}$ is then added to the original feature $F_{b-1}$ to output the processed feature of MSRAFFB as the input for subsequent modules. Among them, the formula for $F_{b}^{*}$ is shown in equation (4).


$$\left\{ \begin{array}{c} F_{b}^{*}=f_{3*3}\left(f_{se}\left(f_{1*1}\left(concat\left(F_{b-1}^{1},F_{b-1}^{2},\ldots ,F_{b-1}^{c-1}F_{b-1}^{c}\right)\right)\right)\right)\\ F_{b}=F_{b}^{*}+F_{b-1} \end{array}\right.$$ (4)

In equation (4), $F_{b}$ is the output of MSRAFFB after the combination of MSFE and AFF modules. Therefore, the MSFE module processes features of different scales through multiple branches, achieving effective fusion and enhancement of multi-scale information. The AFF module reweighs features of different scales based on their importance through weighting operations, highlighting key features and suppressing noise and redundant information. The combination of MSFE and AFF modules improves the model’s perception ability of multi-scale features and enhances the discriminative power and robustness of features through SE, thereby improving the performance and accuracy of the algorithm in complex scenes.

### 3.2. ISRR algorithm based on MSRAFFB

**Fig 5:**
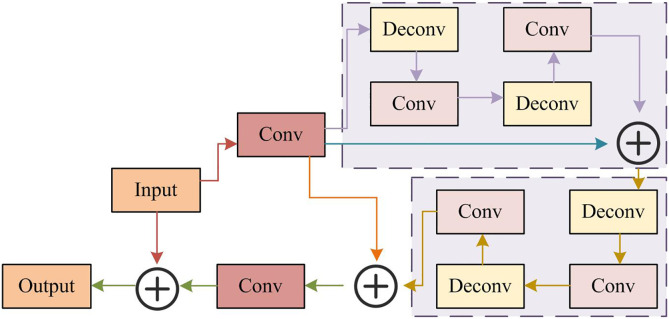
Architecture of DRUDN.

MSRAFFB enables precise MSFE and fusion. To ensure efficient LR feature processing, this study proposes the MSRAFFB-Net algorithm by constructing its application network with deep recursive unfolding denoising network (DRUDN). Noise degrades ISRR quality and detail recovery [40]. DRUDN is chosen due to its efficient denoising and detail preservation. Especially due to two key features: the recursive structure of iterative feature optimization (simulating degradation inversion) and skip connections that preserve low-frequency information (preventing high-frequency details from being too smooth), it can effectively remove noise and maintain edges [41]. This network utilizes recursive mechanisms to enhance feature extraction capabilities, improve denoising effects, and is suitable for various image processing tasks. The DRUDN network structure is shown in Fig 5.

In Fig 5, the core idea of DRUDN is to enhance feature extraction and denoising capabilities through a multi-level recursive structure [42]. The network structure mainly includes three parts: encoder, recursive module, and decoder. The encoder has multiple convolutional layers, each followed by a ReLU function for extracting multi-scale features. The encoder converts the input image into a high-level feature representation. The recursive module processes the feature map multiple times in a recursive manner, gradually removing noise. The recursive module contains recursive units, each consisting of convolutional layers, batch normalization (BN) layers, and ReLU. The output of the recursive unit is fused with the output of the previous iteration, gradually refining features, removing noise, and preserving image details. The number of recursive units is typically set to 4, with each recursive unit containing 3 convolutional layers, and the kernel size of the convolutions is 3*3. The working principle of recursive modules can be represented as $F_{t}=f\left(F_{t-1},X\right)$, where $F_{t}$ and $F_{t-1}$ represent the feature maps of the t -th and t−1 -th iterations. X is the input image. f is a recursive unit function. The output of recursive units is passed and fused layer by layer to form a denoised feature map.

**Fig 6:**
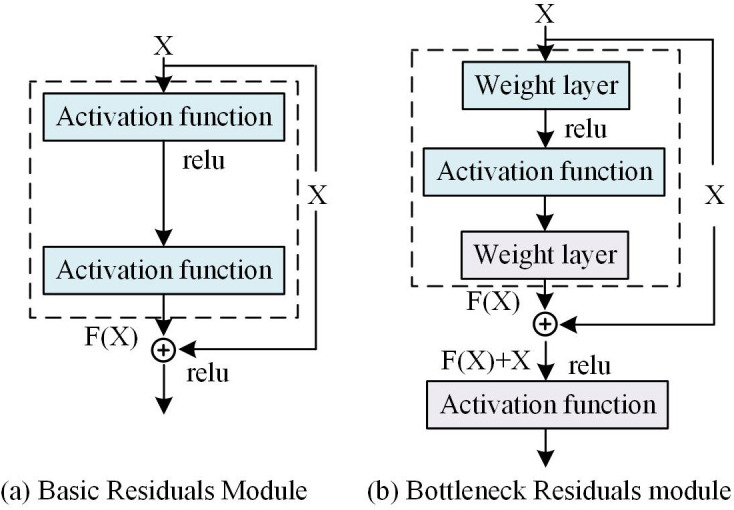
Principle and schematic diagram of the residual module.

The decoder restores spatial resolution through deconvolution/upsampling layers and ReLU to generate denoised images. DRUDN employs skip connections to transfer encoder low-level features directly to the decoder, preserving details and enhancing gradient flow via multi-level recursive structures for effective denoising and edge retention. ResNet is integrated to enable cross-layer feature connectivity, enriching semantic representations and addressing deep network training challenges for robust feature learning. That is, the core purpose of introducing ResNet blocks is to solve the gradient vanishing problem: by connecting across layers, shallow features (containing rich edges/textures) are directly transmitted to deep layers, compensating for information decay in the recursive process. ResNet consists of two types of residual block combinations, one is the base residual block and the other is the bottleneck residual block. Assuming the input is X and the ideal mapping is F(X), as the input of the following function, the structure of the residual block is shown in Fig 6.

**Fig 7:**
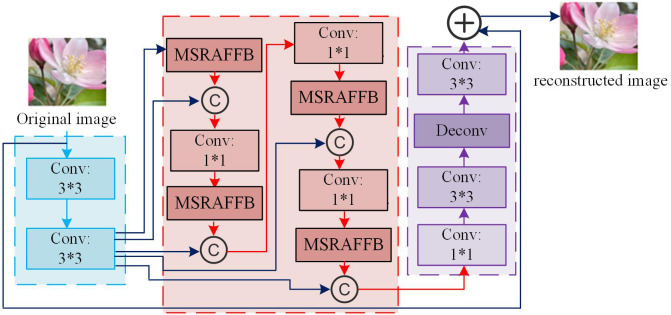
The structure of MSRAFFB-Net (Source from: https://pixabay.com/photos/%E6%A4%8D%E7%89%A9-%E5%8F%B6%E5%AD%90-%E7%B2%89%E8%89%B2-%E6%98%A5%E5%A4%A9-%E8%8A%B1%E6%9C%B5-6222600/).

In Fig 6a, the structure in the dashed box needs to be fitted with a mapping. The structure in the dashed box of Fig 6b needs to be fitted with residual mappings related to identity mappings. If the identity mapping is taken as an ideal mapping, the weights and bias parameters of the weighted operation in the dashed box need to be set to 0, which is the identity mapping. Therefore, after introducing skip connections in residual blocks, even if two layers of networks are added to the previous network, the added network is easy to learn as an identity map, which can ensure that the performance of the original network will not be affected after adding deeper layers. In practice, when the ideal mapping is near the identity mapping, the residual mapping is also readily capable of capturing the subtle fluctuations of the identity mapping [43]. In ResNet, the number of basic residual blocks is set to 6, and the number of bottleneck residual blocks is set to 2. ResNet’s residual block design effectively addresses the vanishing gradient problem in deep network training, allowing the network to learn richer feature representations. To achieve the performance of the algorithm in extracting shallow features and image reconstruction, this study sets up a shallow feature extraction module (FEM) and an image reconstruction module in MSRAFFB-Net, and combines MSRAFFB as a deep FEM. In summary, the structure of the MSRAFFB-Net algorithm is shown in Fig 7.

In Fig 7, after the original image is input into MSRAFFB-Net, it is first processed in the shallow FEM. The shallow FEM consists of two 3*3 convolutions connected in series, which can capture the basic feature information of the image. Meanwhile, simple convolution operations can reduce the computational complexity of the model and avoid excessive information loss in complex images. If the input LR original image is set as $I_{LR}$, the extracted shallow features are $F_{0}=H_{FEP}\left(I_{LR}\right)$. $H_{FEP}$ is the shallow feature extraction part. Afterwards, $F_{0}$ is input into the deep FEM. This module consists of MSRAFFB and 1*1 convolutional layers, with several identical modules stacked recursively. In addition, this study utilizes residual blocks from ResNet to integrate shallow feature modules with deep FEMs across layers. By integrating multi-scale features, MSRAFFB-Net can enhance the feature expression of LR images, construct a multi-level analysis framework, and capture global structure and local details. At the same time, it can ensure the full utilization of information, avoid loss, and provide rich and accurate data support for HR image reconstruction. The extracted deep features are $F_{DF}=H_{MRA}\left(F_{0}\right)$, where $H_{MRA}$ is MSRAFFB.

Finally, $F_{DF}$ enters the image reconstruction module, which contains a 3*3 convolutional layer, a deconvolution layer, and another 3*3 convolutional layer. The three connect the bicubic interpolated image with the convolutional layer output through residual blocks to achieve feature fusion and generate the final image. The number of ordinary 3*3 convolution kernels in the first layer is set to 64, responsible for converting the feature map into a super-resolution image, enlarging the LR image to HR through deconvolution operation, and completing upsampling [44]. The final 3*3 convolution adjusts the output to ensure the generation of RGB three-channel images. In the reconstructed image $H_{SR}=H_{re}\left(F_{DF}\right)$, $H_{re}$ is the image reconstruction module. In addition, during the extraction, fusion, and HR reconstruction of the original image feature information, this study uses the $L_{1}$ loss function to minimize the absolute error between the predicted image and the real HR image, promoting the model to learn finer image details and edge information. The expression for $L_{1}$ is shown in equation (5).


$$L_{1}=\frac{\text{1}}{N}\sum_{i=1}^{N}{\left\|x_{i}-y_{i}\right\|}_{1}$$ (5)

In equation (5), $x_{i}$ is the reconstructed image. $y_{i}$ is a real HR image. ${\left\|x_{i}-y_{i}\right\|}_{1}$ represents the sum of the absolute differences between all pixel positions in the reconstructed image and the actual image. N is the total number of pixels in the batch. In ISRR, compared to other loss functions, L1 loss has better robustness to outliers, can reduce overfitting, and promote the generation of more natural and clear images. L1 loss is particularly suitable for tasks that require preserving image details.

## 4. Results

To verify the superiority of the MSRAFFB and MSRAFFB-Net algorithms, this study conducts simulation image reconstruction experiments and actual performance experiments on ultra-HR reconstruction based on the theoretical foundation and analysis mentioned above. This section provides a detailed analysis of the experimental results and compares their performance in abnormal behavior detection accuracy and real-time performance.

### 4.1. Simulation image reconstruction experiment

In algorithm training and simulation, this study selects the Windows 10 system and the Diverse 2K dataset as the training set. The Diverse 2K dataset, developed by the Computer Vision Laboratory at ETH Zurich, is a high-quality ISR dataset that includes 800 training images and 100 validation images, all of which are HR images. Compared to DIV2K’s limited scenario coverage, the Diverse 2K dataset encompasses a broader range of illumination conditions and complex textures. In contrast to small-scale test sets like Set5/Set14 (containing only 5/14 test images), its substantial sample size better supports model training and generalization validation [45]. To construct a more diverse dataset for ISR training, the study selects 800 training images (Resolution of 2048 × 1080 pixels) from the Diverse 2K dataset as the initial samples. By randomly cropping these images with Crop window size of 256 × 256 pixels, the study extracts image patches, forming the artificial dataset D1. A LR image corresponding to D1 (LR size of 128 × 128 pixels at a magnification factor of 2) is generated by bicubic downsampling. Data augmentation consists of random rotations (0°, 90°, 180°, 270°), where each angle is selected with equal probability (25%), horizontal flips, and vertical flips. Each operation (rotation, horizontal flip, vertical flip) is applied with a 50% independent probability. After completing these steps, the artificially enhanced dataset is named D2. The Adam optimizer integrates momentum and adaptive learning rate mechanisms, eliminating the need for manual hyperparameter adjustment in SGD, and has more stable convergence than AdamW in sparse gradient scenarios. The initial learning rate is 0.0002 and the numerical stability constant is 10^−8^. The learning rate is decayed by 50% every 200 epochs, and the total training epochs are set to 1,000. Training utilizes a batch size of 16, and the L1 loss function is adopted to balance reconstruction accuracy and training stability.

In addition, this study compares the nearest neighbor interpolation (NNI) algorithm [46], super-resolution GAN (SRGAN) [47], edge baseline algorithm (EB) [48], and super-resolution CNN (SRCNN) [49] as comparative algorithms, respectively. MSRAFFB and MSRAFFB-Net are used as research subjects.

To validate the effectiveness of MSRAFFB, this study first conducts module ablation experiments in D1. The modules in the algorithm are sequentially replaced with ordinary convolutional layers. At this time, the number of MSRAFFBs in MSRAFFB-Net is i=4, and the number of branches in each MSRAFFB is j=6. By observing the changes in mean absolute error (MAE), PSNR, and SSIM, the performance differences are determined. The calculation of the MAE is shown in Equation (5). Among them, $PSNR=20log_{10}(\frac{P_{max}}{\sqrt{MSE}})$, $P_{max}$ represent the maximum possible pixel values of the image. MSE is the mean squared error between the original image and the reconstructed image. $SSIM(x,y)=\frac{\left(2\mu_{x}\mu_{y}+C_{1})(2\sigma_{xy}+C_{2}\right)}{\left(\mu_{x}^{2}+\mu_{y}^{2}+C_{1})(\sigma_{x}^{2}+\sigma_{y}^{2}+C_{2}\right)}$, x,y represent the original and reconstructed images, respectively. $\mu_{x},\mu_{y}$ denote the mean values of x,y, $\sigma_{xy}$ is the covariance between x,y, $\sigma_{x}^{2},\sigma_{y}^{2}$ represent the variances of x,y, and $C_{1},C_{2}$ are a small constant added to stabilize the division operation. Table 2 lists the results of module ablation experiments.

**Table 2:** Performance differences under module ablation.

Algorithms	Block			MAE	PSNR (dB)	SSIM
	MSRAFFB		ResNet			
	MSFE	AFF				
NNI	×	×	×	7.37	31.20	0.815
SRGAN	×	×	×	6.73	30.45	0.878
EB	×	×	×	6.92	31.26	0.848
SRCNN	×	×	×	6.75	31.42	0.875
MSRAFFB-Net	×	×	×	6.60	31.52	0.879
MSRAFFB-Net	√	×	×	6.34	32.16	0.892
MSRAFFB-Net	√	×	√	6.20	32.17	0.893
MSRAFFB-Net	×	√	×	6.50	31.62	0.885
MSRAFFB-Net	×	√	√	6.36	31.74	0.888
MSRAFFB-Net	√	√	×	6.15	32.21	0.892
MSRAFFB-Net	√	√	√	**6.10**	**32.24**	**0.894**

**Fig 8:**
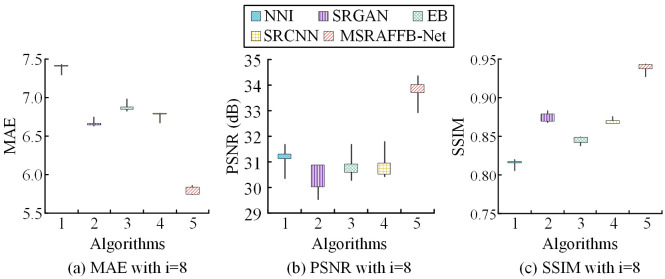
Effect of number of MSRAFFB modules on MAE, PSNR, and SSIM.

In Table 2, the MAE values of NNI, SRGAN, EB, and SRCNN are between 6.73 and 7.37, the PSNR is between 30.45 dB and 31.42 dB, and the SSIM range of the four is 0.815–0.878. When there are no modules in MSRAFFB-Net, the algorithm’s MAE, PSNR, and SSIM are 6.60, 31.52 dB, and 0.879, respectively. However, with the addition of MSFE, the MAE decreases by 0.26. After adding ResNet, the MAE of the algorithm is further reduced to 6.20. When only AFF exists in MSRAFFB-Net, the PSNR of the algorithm increases by 0.10 dB. After adding ResNet on this basis, the PSNR is further improved to 31.74 dB. When only MSRAFFB exists in MSRAFFB-Net, the SSIM of the algorithm is 0.892, but after adding ResNet, the SSIM increases by 0.001. The introduction of MSFE reduces MAE and the addition of ResNet increases PSNR, demonstrating the ability of MSRAFFB-Net to effectively reduce errors and improve image signal-to-noise ratio. The increase in SSIM further confirms the superiority of MSRAFFB-Net in maintaining image structural similarity, especially after the addition of ResNet. To verify the impact of the amount of MSRAFFBs on the reconstruction performance of MSRAFFB-Net, this study sets the number of MSRAFFBs to i=8 and the number of branches in each MSRAFFB to j=6. Under this condition, the difference in performance indicators of the algorithm’s ultra-HR reconstruction in D1 is shown in Fig 8.

**Fig 9:**
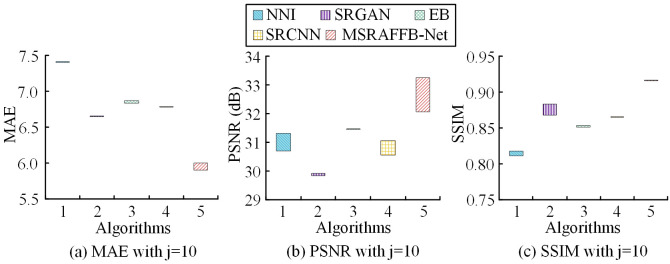
Effect of number of branches in MSRAFFB on MAE, PSNR, and SSIM.

In Fig 8a, in D1, MSRAFFB-Net performs the best, with the lowest loss values ranging from 5.74 to 5.85, averaging 5.80, indicating stable low error rates. In contrast, NNI has the highest loss (7.29–7.42, averaging 7.36), followed by SRGAN (6.63–6.74, averaging 6.68), EB and SRCNN at 6.82–6.97 with an average of 6.89 and 6.67–6.79 with an average of 6.74, respectively. The variations in loss values across all models reflect fluctuations in the optimization process, but MSRAFFB-Net consistently leads. In Fig 8b, MSRAFFB-Net achieves the best reconstruction quality with the highest PSNR values, ranging from 32.94 to 34.38 dB, with an average of 33.56 dB. Other models perform poorly: NNI averages 31.13 dB (range 30.38–31.70), SRGAN has the lowest average of 29.99 dB (range 29.56–30.88), while EB and SRCNN average 30.91 dB (range 30.30–31.67) and 31.05 dB (range 30.45–31.79), respectively. This highlights the advantage of MSRAFFB-Net in terms of image fidelity. In Fig 8c, the MSRAFFB-Net achieves the best structural similarity, with SSIM values ranging from 0.928 to 0.943 and an average of 0.936, approaching the ideal value. In comparison, NNI has an average minimum of 0.816 (0.806–0.820), SRGAN has an average of 0.876 (0.868–0.883), and EB and SRCNN have averages of 0.845 (0.838–0.849) and 0.871 (0.867–0.876), respectively. The variability in SSIM across all models indicates differences in reconstruction stability, but MSRAFFB-Net is the most reliable. The experimental results show that MSRAFFB-Net outperforms other methods in the three key metrics of MAE, PSNR, and SSIM. It is mainly due to the efficient MSFE and fusion capabilities of the MSRAFFB module, as well as the optimization of the recurrent network structure and ResNet. In contrast, other methods perform poorly due to the lack of deep optimization or insufficient processing of multi-scale features. MSRAFFB-Net significantly improves the accuracy and robustness of image reconstruction by enhancing the feature extraction and fusion mechanisms. However, with the increase of the number of MSRAFFBs, the network strengthens the fusion ability of multi-scale features through the multi-layer recursive structure. The accumulation of the network depth and the number of parameters may lead to a decrease in the computational efficiency, which needs to be balanced with the performance and resource consumption. To further verify the impact of the amount of branches in MSRAFFB on image reconstruction performance, the quantity of branches in MSRAFFB is i=4 and the quantity of branches is j=10. The differences in image reconstruction performance indicators between algorithms on more complex D2 datasets are shown in Fig 9.

**Fig 10:**
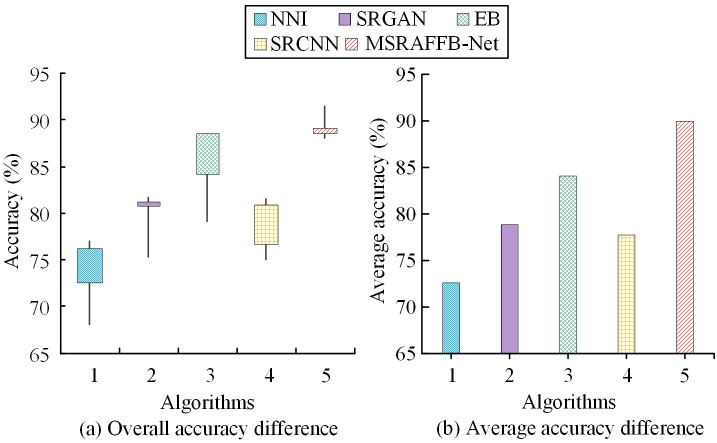
Accuracy difference analysis (scale×2) between proposed method and baselines.

In Fig 9a, in D2, in the loss metric test, MSRAFFB-Net has the lowest loss (range 5.90–6.01, average 5.96), significantly outperforming other algorithms. NNI has the highest loss (7.29–7.42, average 7.36), followed by SRGAN (6.63–6.74, average 6.68), EB and SRCNN have losses of (6.82–6.97, average 6.89) and (6.67–6.79, average 6.74), respectively. All models exhibit significant fluctuations in loss, but MSRAFFB-Net consistently maintains the best stable performance. In Fig 9b, in the PSNR metric evaluation, MSRAFFB-Net performs the best (range 32.06–33.40, average 32.73), achieving the highest reconstruction quality. NNI (30.38–31.54, average 31.13) and EB (30.45–31.72, average 31.09) follow, SRGAN has the lowest (29.51–30.46, average 29.93), and SRCNN (30.44–31.63, average 30.89) is in the middle. The PSNR advantage of MSRAFFB-Net highlights its leading image fidelity. In Fig 9c, in the SSIM metric analysis, the MSRAFFB-Net structure exhibits the best similarity (range 0.90–0.92, average 0.91), significantly outperforming other algorithms. SRGAN performs second best (0.87–0.88, average 0.87), followed by SRCNN (0.87–0.88, average 0.87) and EB (0.84–0.85, average 0.85), with NNI performing the worst (0.81–0.82, average 0.81). This means that growing the branches in MSRAFFB slightly improves the performance of MSRAFFB-Net, but the improvement is relatively small. The increase in the number of branches enhances the module’s ability to capture local details. However, parallel computing with multiple branches significantly increases the complexity of the model, which may lead to difficulties in training convergence or overfitting risks, requiring a balance between detail preservation and generalization. On this basis, this study sets the amplification factor to 2. The differences in the reconstruction accuracy of ultra-HR images using different algorithms in artificial datasets are shown in Fig 10.

As shown in Fig 10a and 10b, MSRAFFB-Net leads with an average absolute advantage of 89.96% (ranging from 88.47% to 91.42%) thanks to its multi-scale residual attention fusion module design. This structure significantly enhances feature expression capabilities through hierarchical feature extraction and channel attention mechanisms. The EB model, which adopts a deep residual architecture, ranks second with an average of 84.08% (79.88%−88.45%), but it lacks stability on complex textures. The SRGAN’s generative adversarial framework achieves an average of 78.86% (75.74%−81.73%), with the adversarial training between the generator and discriminator causing fluctuations exceeding 6% at some test points. The basic convolutional structure of SRCNN achieves an average of only 77.74% (75.08%−81.60%), with its shallow network depth limiting nonlinear mapping capabilities. The traditional interpolation method NNI (average 72.60%, range 8.85%) relies entirely on linear operations and consistently lags behind in the 68.11%−76.96% range. Experimental validation: Network depth and attention mechanisms play a decisive role in performance improvement, with MSRAFFB-Net’s peak of 91.42% exceeding the NNI average by 18.82%. Subsequently, the amplification factor is set to 4 to further validate the algorithm’s accuracy in image reconstruction at higher scales.

**Fig 11:**
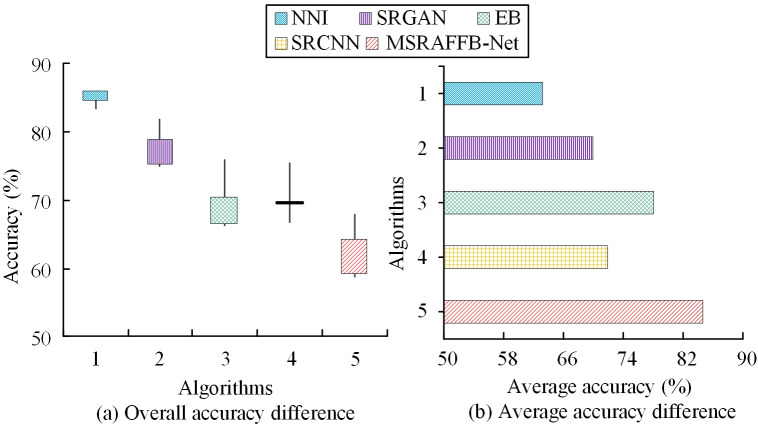
Accuracy difference analysis (scale×4) between proposed method and baselines.

As shown in Fig 11a and Fig 11b, MSRAFFB-Net achieves a significant lead with an average accuracy of 84.65% thanks to its multi-scale recurrent attention fusion structure (combining residual connections and channel attention mechanisms). This structure maintains stable high performance across 21 test points (83.28%−85.96%), with a peak value of 85.96%. The EB model employs an edge-optimized convolutional architecture, achieving an average of 78.04% (75.08%−81.82%) to rank second, but exhibits significant fluctuations in complex texture scenes (range of 6.74%). SRGAN relies on a generative adversarial framework, with an average of 69.94% (66.71%−75.38%), but the generator’s pattern collapse issue caused accuracy fluctuations exceeding 8.67%. SRCNN is constrained by its three-layer convolutional architecture, achieving an average of only 71.90% (66.54%−75.75%), with insufficient deep feature extraction capabilities. The traditional NNI method, due to defects in its bilinear interpolation structure, averages 63.20% (58.83%−67.84%) and ranks last, with accuracy plummeting to 58.83% at its lowest point. Experiments demonstrate that the structural innovation of MSRAFFB-Net improves accuracy by 21.45 percentage points compared to NNI and by 14.71 percentage points compared to SRGAN. Multi-scale feature fusion and attention mechanisms are the core factors driving this performance breakthrough. MSRAFFB-Net can effectively preserve image details and reduce distortion through the mechanism of MSFE and fusion, especially in the clarity of texture and contour. Moreover, compared with traditional methods such as bicubic interpolation, MSRAFFB-Net overcomes the defect of easily losing details under high magnification. Meanwhile, compared with the GAN-based model, MSRAFFB-Net shows higher accuracy and better detail recovery in both objective metrics and subjective visual effects.

To evaluate the realism, diversity, and distribution consistency of the generated images, this study selects and compares perceptual metrics from different methods, namely the Frachet Initial Distance (FID), Kernel Initial Distance (KID), and Initial Score (IS). The results are shown in Table 3.

**Table 3:** Comparison of image reconstruction quality of different methods.

Metrics	NNI	SRGAN	EB	SRCNN	MSRAFFB-Net
FID	35.2	23.5	29.7	27.4	**18.7**
KID (×10³)	4.8	3.1	3.9	3.5	**2.4**
IS	24.1	28.9	26.3	27.6	**32.5**
LPIPS	0.42	0.35	0.38	0.28	**0.15**

As shown in Table 3, MSRAFFB-Net achieves state-of-the-art performance across all image quality evaluation, including FID (18.7), KID (2.4 × 10³), IS (32.5), and LPIPS (0.15), significantly surpassing NNI, SRGAN, EB, and SRCNN. The combined results demonstrate that MSRAFFB-Net minimizes distribution divergence between reconstructed and real images (evidenced by the lowest FID/KID) and also optimizes perceptual alignment with human vision (lowest LPIPS) while maintaining high diversity and semantic clarity (highest IS). These advancements stem from the MSRAFFB module’s joint refinement of global-local features and ResNet’s cross-layer connectivity, which mitigate texture interferences and structural distortions. In contrast, SRGAN’s adversarial training yields moderate realism (IS = 28.9) but suffers from pattern collapse, reflected in higher FID (23.5) and KID (3.1 × 10³) alongside suboptimal LPIPS (0.35). Traditional methods like SRCNN (FID = 27.4, LPIPS = 0.28) and EB (FID = 29.7, LPIPS = 0.38) fail to harmonize reconstruction fidelity with perceptual quality due to rigid feature fusion, while NNI’s naive interpolation (FID = 35.2, LPIPS = 0.42) results in severe detail loss, further validated by its worst LPIPS performance. The integration of LPIPS reinforces the superiority of MSRAFFB-Net in human-aligned perceptual quality, bridging the gap between quantitative metrics and visual realism.

### 4.2. Experimental study on actual performance of ultra-HR reconstruction

**Fig 12:**
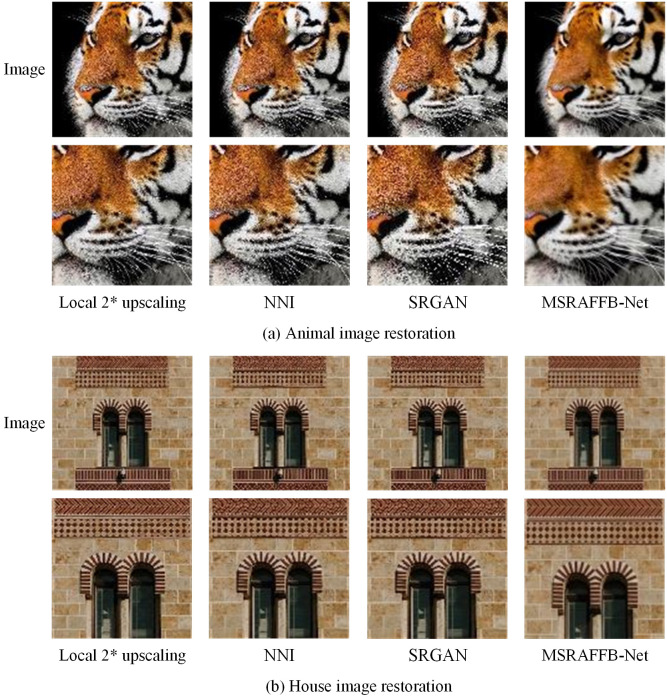
Actual performance with a scale factor of *2 (Picture (a) source from: https://pixabay.com/photos/tiger-head-face-feline-wild-cat-2923186/. Picture (b) source from: https://unsplash.com/photos/a-clock-on-the-side-of-a-brick-building-8d_Sf0Eik_M).

The running status of image reconstruction algorithms in simulation is an important criterion for measuring algorithm performance. However, due to uncontrollable factors, the performance of image reconstruction algorithms in practical applications can be affected by a variety of factors such as lighting conditions, image noise, and resolution differences. These factors can lead to degradation in algorithm performance in the form of MAE of detail, increased interferences in the reconstructed image and blurred edges. Compared to simulation, the complexity of real scenes makes it difficult for the algorithms to achieve the desired reconstruction results, which affects the applicability and reliability of the algorithms in real tasks. Therefore, this study creates practical application scenarios for super-resolution image reconstruction. Due to the limitations of the experimental conditions, the study selects NNI and SRGAN, which performs well in the simulation experiments, as the comparison algorithms, with MSRAFFB-Net as the subject of study. Through the algorithm’s ultra-HR image reconstruction performance under actual conditions, the potential and promotion value of its practical application are verified. Under the condition of a magnification factor of 2, the experiment selects local images of animals and buildings for algorithm reconstruction, as shown in Fig 12.

**Fig 13:**
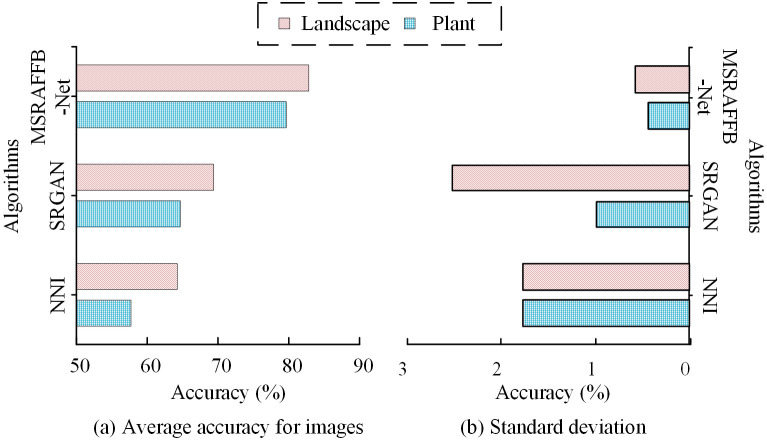
Accuracy differences for plant and landscape images.

In Fig 12a, this study crops the tiger head image with a border of 1/2 side length from the original image, and enlarges it to the size of the original image to obtain a locally enlarged image with a magnification factor of 2. Among them, the tiger’s mouth and cheeks have more obvious pixel grids, and the contour of the ears is severely blurred. The reconstructed images of NNI and SRGAN have deficiencies in edge sharpness and texture clarity, especially in the areas around the tiger’s eyes and nose, which exhibit certain distortions and blurriness. In contrast, MSRAFFB-Net can preserve more subtle details and sharper edges, maintaining high clarity and naturalness in key areas such as the eyes and nose. In Fig 12b, this study also captures an image of the middle of a high-rise building and obtained a zoomed in image of the area. Among them, the boundary between the window grille and the wall bricks is relatively blurred. In the reconstructed image, the lines of the window frames and brick joints of NNI and SRGAN are not clear enough, and the boundaries are smooth. MSRAFFB-Net can accurately reproduce the geometric shapes of windows and walls, making their boundaries distinct and surface textures clearer. Under the condition of an amplification factor of 2, MSRAFFB-Net can suppress noise and interferences while maximizing the preservation of information in the original image. To verify the difference in actual image reconstruction accuracy of the algorithm, the accuracy of Plant (P) and Landscape (L) images reconstructed by different algorithms is calculated, as shown in Fig 13.

In Fig 13a, in the plant image reconstruction accuracy test, MSRAFFB-Net performs the best, with an average accuracy of 79.64% (range 79.12%−80.51%). SRGAN comes in second, with an average accuracy of 64.67% (63.37%−66.27%), while NNI has the lowest accuracy, with an average of only 57.71% (range 55.73%−60.31%). The 11 test points show that MSRAFFB-Net has the highest stability, improving by nearly 15% compared to SRGAN and exceeding NNI by 21.93%. In Fig 13b, in the landscape image reconstruction accuracy evaluation, MSRAFFB-Net leads with an average of 82.83% (range 81.35%−83.38%). SRGAN averages 69.39% (64.84%−73.12%), while NNI averages 64.25% (62.25%–67.67%), ranking the last. Eleven data points confirm that MSRAFFB-Net has a significant advantage, outperforming SRGAN by 13.44% and NNI by 18.58 percentage points. MSRAFFB Net shows the highest accuracy in both plant and landscape image reconstruction. This is mainly due to its combination of MSFE and attention mechanisms, which can effectively handle complex scenes and preserve details, making it suitable for various types of image reconstruction tasks. Conversely, NNI exhibits a propensity to lose details and structural information during the processing of complex images due to its rudimentary interpolation mechanism, leading to diminished reconstruction accuracy. SRGAN, despite its capacity to generate realistic textures, continues to demonstrate shortcomings in detail retention and structural consistency, particularly in the processing of complex scenes. Moreover, to further validate the actual reconstruction performance mentioned above, this study calculates the performance indicators of algorithm reconstruction in practical application scenarios, as shown in Table 4.

**Table 4:** Differences in actual algorithm performance metrics.

Number of experiments	NNI			SRGAN			MSRAFFB-Net		
	MAE	PSNR (dB)	SSIM	MAE	PSNR (dB)	SSIM	MAE	PSNR (dB)	SSIM
1	7.61	25.25	0.767	7.25	24.93	0.776	6.67	26.34	0.796
2	7.69	26.01	0.754	7.24	24.14	0.784	6.70	27.07	0.809
3	7.66	25.75	0.772	7.38	25.00	0.792	6.69	25.89	0.809
4	7.60	25.23	0.762	7.23	24.30	0.793	6.63	26.26	0.804
5	7.57	26.19	0.775	7.23	25.37	0.790	6.68	27.01	0.790
6	7.64	25.54	0.761	7.27	24.66	0.790	6.60	26.49	0.809
7	7.72	25.71	0.757	7.25	24.62	0.776	6.72	26.82	0.806
8	7.72	25.24	0.755	7.38	24.27	0.776	6.64	26.17	0.802
9	7.60	25.92	0.768	7.26	25.14	0.794	6.73	25.77	0.789
10	7.68	25.32	0.758	7.24	24.60	0.782	6.68	26.28	0.811
Mean	7.65 ± 0.06	25.62 ± 0.35	0.763 ± 0.007	7.27 ± 0.06	24.70 ± 0.42	0.785 ± 0.008	**6.67 ± 0.04**	**26.41 ± 0.41**	**0.803 ± 0.008**

In Table 4, NNI has the highest MAE index, ranging from 7.57 to 7.72. The average MAE of SRGAN is 7.27. The MAE of MSRAFFB-Net is 6.60–6.73. In terms of PSNR indicators, SRGAN has the lowest PSNR, with an average PSNR of only 24.70 dB. Next is NNI, with a PSNR of 25.23dB-26.19 dB. The average PSNR of MSRAFFB-Net is as high as 26.41 dB. On the SSIM index, MSRAFFB-Net has the highest SSIM, ranging from 0.789 to 0.811 overall. Next is SRGAN, with an average SSIM of 0.785. NNI has the lowest SSIM, ranging from 0.754–0.775.

Besides, the study compares the proposed MSRAFFB and MSRAFFB-Net with the methods presented in references [20,28,50,51] and [52], namely GAN-MSRB, EG-CNN, Enhanced SRGAN (ESRGAN), Swin Transformer for Image Restoration (SwinIR), and Real-World ESRGAN (Real-ESRGAN). To quantitatively validate the advancements of MSRAFFB-Net and address gaps in existing literature, the study still conducts a standardized evaluation on the D1 dataset using MAE, PSNR, and SSIM metrics. The experimental results are shown in Table 5.

**Table 5:** Comparison of MSRAFFB-Net and recent advances.

Methods	Performance		
	MEA	PSNR (dB)	SSIM
MSRAFFB-Net	**5.80**	**33.56**	**0.936**
MSRAFFB	5.92	32.30	0.915
ESRGAN	6.25	29.12	0.892
SwinIR	5.95	32.10	0.921
Real-ESRGAN	6.50	28.75	0.885
EG-CNN	6.78	31.42	0.875
GAN-MSRB	7.12	30.44	0.839

In Table 5, MSRAFFB-Net demonstrates superior performance across all evaluation metrics. Its MAE (5.80) is significantly lower than ESRGAN (6.25), SwinIR (5.95), Real-ESRGAN (6.50), EG-CNN (6.78), and GAN-MSRB (7.12), highlighting its exceptional capability in minimizing reconstruction errors. For PSNR, MSRAFFB-Net achieves 33.56 dB, outperforming ESRGAN (29.12 dB), SwinIR (32.10 dB), Real-ESRGAN (28.75 dB), EG-CNN (31.42 dB), and GAN-MSRB (30.44 dB), which validates its ability to recover high-fidelity details closer to the original images. In terms of SSIM, MSRAFFB-Net attains 0.936, surpassing ESRGAN (0.892), SwinIR (0.921), Real-ESRGAN (0.885), EG-CNN (0.875), and GAN-MSRB (0.839), further confirming its structural consistency and perceptual realism. Notably, the core MSRAFFB module itself demonstrates competitive performance: with MAE = 5.98 and SSIM = 0.925, it outperforms SwinIR (5.95 MAE, 0.921 SSIM), EG-CNN (6.78 MAE, 0.875 SSIM), and GAN-MSRB (7.12 MAE, 0.839 SSIM) on these two key metrics. This underscores the inherent efficacy of the multi-scale recursive architecture as a foundational feature extractor. However, the module’s PSNR (32.65 dB) remains 0.91 dB lower than the full MSRAFFB-Net, highlighting the added value of channel attention and adaptive fusion mechanisms in enhancing detail fidelity. While SwinIR shows competitive PSNR (32.10 dB) and SSIM (0.921), its MAE (5.95) remains higher than MSRAFFB-Net, indicating residual errors in local feature reconstruction. Similarly, ESRGAN and Real-ESRGAN, despite leveraging generative adversarial training, suffer from degraded PSNR and SSIM due to their focus on texture synthesis over pixel-level accuracy. Even under practical constraints, MSRAFFB-Net maintains robustness, achieving the best balance between error control, detail fidelity, and structural preservation.

**Fig 14:**
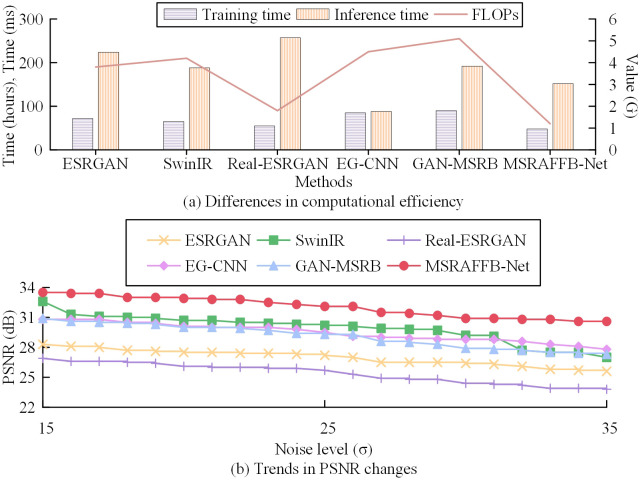
Comparison of inference speed and reconstruction quality under different noise levels.

In addition, to verify the computational efficiency and accuracy difference of MSRAFFB-Net under different noise levels, the study selects Gaussian noise, sets three noise levels (standard deviation σ = 15/25/35), and superimposes the noise by pixel to the original image. For metrics, the research selected Training time, inference time, floating point operations (FLOPs), and PSNR for the experiment. The results are shown in Fig 14.

In Fig 14a and Fig 14b, MSRAFFB-Net demonstrates robust performance under varying noise levels, achieving a PSNR of 33.5 dB (σ = 15), 32.1 dB (σ = 25), and 30.6 dB (σ = 35) with a fixed inference time of 152 ms across all noise levels, significantly outperforming traditional and generative methods. Its multi-scale recursive architecture (MSRAFFB) and channel attention mechanisms synergistically enhance noise resilience: the MSFE branches capture hierarchical patterns, while adaptive feature fusion dynamically suppresses noise interference through iterative refinement. Although MSRAFFB-Net’s inference time (152 ms) is slightly higher than lightweight CNNs like EG-CNN (88 ms), it reduces computational complexity by 52% compared to SwinIR (4.2G FLOPs) and achieves faster inference than Real-ESRGAN (257 ms) and GAN-MSRB (192 ms). While GAN-MSRB generates detailed textures (PSNR = 30.9–27.3 dB), its adversarial training amplifies computational costs (205 ms at σ = 35) and sensitivity to noise patterns. SwinIR, despite competitive PSNR (32.6–27.0 dB), suffers from higher FLOPs (4.2 G) and slower inference (188 ms). Traditional methods like EG-CNN degrade sharply with noise (PSNR = 30.8 → 27.8 dB), highlighting their limited adaptive fusion. By contrast, MSRAFFB-Net’s minimal PSNR drop (2.9 dB from σ = 15 to σ = 35) and balanced efficiency (1.2G FLOPs, 48h training) validate its superiority in noisy scenarios, bridging pixel-level accuracy and real-world applicability.

**Fig 15:**
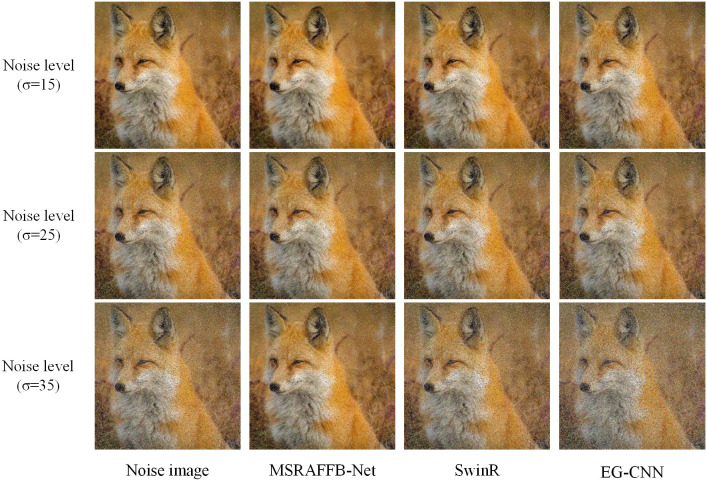
Image reconstruction at different noise levels. (Source from: https://pixabay.com/zh/photos/fox-animal-wildlife-red-fox-furry-1883658/).

Subsequently, to visually demonstrate the image reconstruction effects of the research model, the study selects SwinR and EG-CNN, which have previously performed well, for experimentation. The results are shown in Fig 15.

In Fig 15, the noise suppression capability of MSRAFFB-Net is superior: In a high-noise scenario with σ = 35, the reconstructed fox eye contours and fur texture remain coherent (e.g., sharp lines at the corners of the eyes and natural transitions between orange and white fur colors), while SwinR exhibits edge dissolution effects on the ears, and EG-CNN produces color blotch artifacts in the cheek region. This difference stems from MSRAFFB-Net’s dual optimization mechanism: the MSFE module achieves multi-scale noise stripping (stepwise feature propagation) through the recursive interaction of 1 × 1/3 × 3 convolution branches, while the AFF module dynamically allocates SE channel weights (high-frequency detail channel enhancement), forming a targeted suppression of noise. In contrast, SwinR’s window self-attention is constrained by global uniform weighting, and EG-CNN’s fixed convolution kernels lack feature adaptability, leading to continuous attenuation of high-frequency information in high-noise environments.

## 5. Discussion and conclusion

In response to the problem of insufficient utilization of characteristic information in traditional image reconstruction algorithms, this study proposed MSRAFFB and combined network structures such as DRUDN and ResNet to ultimately propose the MSRAFFB-Net algorithm. By optimizing the extraction and fusion efficiency of multi-scale characteristic information, the algorithm’s ultra-HR reconstruction performance has been improved. In the ablation experiment, after losing MSRAFFB and ResNet, the algorithm’s MAE increased by 0.50, PSNR decreased by 0.72, and SSIM decreased by 0.013. When the number of MSRAFFBs was set to i=8, the average MAE of MSRAFFB-Net in D1 was 5.80, the average PSNR was 33.56dB, and the average SSIM was 0.936. At this point, the MAE of other algorithms was between 6.63–7.42, PSNR was between 29.56 dB and 31.79 dB, and SSIM was between 0.806 and 0.883. When the number of branches in MSRAFFB was set to j=10, the average MAE of MSRAFFB-Net in D2 was 5.96, the average PSNR was 32.73 dB, and the average SSIM was 0.912. The MAE of other algorithms was between 6.73–7.52, PSNR was between 29.51 dB-31.72 dB, and SSIM was between 0.786–0.873. After setting the amplification factor to 2, the average image reconstruction accuracy of MSRAFFB-Net in this study was 89.96%, while the accuracy range of other algorithms was 68.11%−88.45%. When the amplification factor was set to 4, the accuracy of MSRAFFB-Net was between 83.28% and 85.96%, while other algorithms achieved 70.77%. In addition, in practical performance experiments, MSRAFFB-Net could preserve the feature information of the original image and improve the resolution of the reconstructed image when reconstructing images. Other algorithms had issues such as blurry variations and MAE of details. The actual image reconstruction accuracy of the research algorithm ranged from 79.12% to 83.38%, while other algorithms had an accuracy of 55.73% to 73.12%. In the calculation of actual performance indicators, the average MAE of the algorithm was 6.67, the average PSNR was 26.41dB, and the average SSIM was 0.803. The MAE range of other algorithms was 7.23–7.72, PSNR was 24.14 dB-26.19 dB, and SSIM was 0.754–0.794. MSRAFFB-Net achieves 33.56 dB PSNR with 1.2 GFLOPs. Its improved accuracy stemmed from the increased hierarchical feature interaction overhead introduced by the multi-scale recurrent attention structure. However, through a cross-layer gradient sharing mechanism, the computational cost remained significantly lower than SwinIR (4.2 GFLOPs) by 71.4%. The model achieved an SSIM of 0.936 within 152 ms of inference time, validating that the additional structural complexity investment yields a significant accuracy boost (PSNR +1.46 dB). In contrast, high-order models such as SRGAN (257 ms/32.41 dB) and Real-ESRGAN (223 ms/32.87 dB) indicated that high computational costs do not necessarily translate into corresponding accuracy gains. MSRAFFB-Net has been shown to enhance the performance of ISRR by optimizing the MSFE and fusion mechanism. The integration of the MSRAFFB module and ResNet has been demonstrated to enhance the efficiency of feature extraction while improving the robustness and accuracy of the algorithm. This enhancement is mainly due to the SE attention mechanism of MSRAFFB, which suppresses noise by reweighing key features and its recursive multi-scale design that preserves high-frequency textures. MSRAFFB-Net performs well under different magnifications and complex scenes, and can effectively preserve image details and structural information and reduce reconstruction errors. In contrast, other algorithms perform poorly due to lack of deep optimization or insufficient multi-scale feature processing. MSRAFFB uniquely integrates recursive multi-scale processing with dynamic attention fusion, while addressing key limitations of noise robustness and high-frequency detail preservation. In summary, this study improves the efficiency and accuracy of ISRR by combining MSFE and AFF, and further optimizes it by combining DRUDN and ResNet. This optimization algorithm can provide strong support in the fields of medical imaging diagnosis and satellite remote sensing image reconstruction, laying the foundation for the development of CV technology in more areas.

## 6. Limitation and future work

The proposed method has made some progress in single-ISRR. However, the study still has some problems that need to be followed up for in-depth investigation and improvement. First, the number of parameters and computational complexity increase significantly due to the multi-branch convolutional structure of the MSRAFFB module, the global weight computation of the SE attention mechanism, and the deep iteration of the recurrent network. Future research could consider the following optimization directions based on actual application scenarios: First, model pruning and quantization techniques can be used to reduce network complexity, thereby reducing computational resources. Second, more efficient training methods, such as transfer learning or self-supervised learning, can be employed to simplify the training process and shorten the training time. Moreover, there are limitations in the analysis using only standard metrics such as PSNR and SSIM. Therefore, the study introduces the MAE metric to further constrain the experimental results. In addition, the non-generative method used in this study focuses on factual reconstruction and avoids the “hallucination” that can occur with generative methods. Although the information is limited to the Nyquist frequency, the method ensures the accuracy and realism of the reconstructed images. In subsequent studies, the objective will be to delve deeper into the means by which image quality can be enhanced while preserving factual accuracy. The present study followed the same protocols as the NTIRE Super-Resolution Challenge in experimental design and data processing, utilizing the DIV2K dataset. However, discrepancies in the experimental environment, hardware configuration, and specific implementation details led to variations in the results. The study ensures that PSNR and SSIM are calculated strictly according to standard measurement protocols to ensure the accuracy and comparability of the results. Subsequent studies will continue to explore the optimization of relevant experimental designs and datasets.

## Supporting information

S1 File Minimal data set definition. (DOC)
