# Supplementary material for: Image super-resolution reconstruction algorithm based on multi-scale recursive attention and feature fusion
Source: PLoS One. 2025 Oct 7;20(10):e0333398. doi: 10.1371/journal.pone.0333398 (PMC12503264; doi:10.1371/journal.pone.0333398)
Supplement: S1 File — (DOC) [file pone.0333398.s001.doc]

**The data in Table 2**

| Algorithms | Block | | | Loss | PSNR（dB） | ssim |
| --- | --- | --- | --- | --- | --- | --- |
|  | MSRAFFB | | ResNet |  |  |  |
|  | MSFE | AFF |  |  |  |  |
| M1 | × | × | × | 7.37 | 31.20 | 0.815 |
| M2 | × | × | × | 6.73 | 30.45 | 0.878 |
| M3 | × | × | × | 6.92 | 31.26 | 0.848 |
| M4 | × | × | × | 6.75 | 31.42 | 0.875 |
| MSRAFFB-Net | × | × | × | 6.60 | 31.52 | 0.879 |
| MSRAFFB-Net | √ | × | × | 6.34 | 32.16 | 0.892 |
| MSRAFFB-Net | √ | × | √ | 6.20 | 32.17 | 0.894 |
| MSRAFFB-Net | × | √ | × | 6.50 | 31.62 | 0.885 |
| MSRAFFB-Net | × | √ | √ | 6.36 | 31.74 | 0.888 |
| MSRAFFB-Net | √ | √ | × | 6.15 | 32.21 | 0.892 |
| MSRAFFB-Net | √ | √ | √ | 6.10 | 32.24 | 0.893 |

**The data in Table 3**

| metrics | NNI | SRGAN | EB | SRCNN | MSRAFFB-Net |
| --- | --- | --- | --- | --- | --- |
| FID | 35.2 | 23.5 | 29.7 | 27.4 | 18.7 |
| KID (×10³) | 4.8 | 3.1 | 3.9 | 3.5 | 2.4 |
| IS | 24.1 | 28.9 | 26.3 | 27.6 | 32.5 |

**The data in Table 4**

| Number of experiments | NNI | | | SRGAN | | | MSRAFFB-Net | | |
| --- | --- | --- | --- | --- | --- | --- | --- | --- | --- |
|  | Loss | PSNR (dB) | SSIM | Loss | PSNR (dB) | SSIM | Loss | PSNR (dB) | SSIM |
| 1 | 7.61 | 25.25 | 0.767 | 7.25 | 24.93 | 0.776 | 6.67 | 26.34 | 0.796 |
| 2 | 7.69 | 26.01 | 0.754 | 7.24 | 24.14 | 0.784 | 6.70 | 27.07 | 0.809 |
| 3 | 7.66 | 25.75 | 0.772 | 7.38 | 25.00 | 0.792 | 6.69 | 25.89 | 0.809 |
| 4 | 7.60 | 25.23 | 0.762 | 7.23 | 24.30 | 0.793 | 6.63 | 26.26 | 0.804 |
| 5 | 7.57 | 26.19 | 0.775 | 7.23 | 25.37 | 0.790 | 6.68 | 27.01 | 0.790 |
| 6 | 7.64 | 25.54 | 0.761 | 7.27 | 24.66 | 0.790 | 6.60 | 26.49 | 0.809 |
| 7 | 7.72 | 25.71 | 0.757 | 7.25 | 24.62 | 0.776 | 6.72 | 26.82 | 0.806 |
| 8 | 7.72 | 25.24 | 0.755 | 7.38 | 24.27 | 0.776 | 6.64 | 26.17 | 0.802 |
| 9 | 7.60 | 25.92 | 0.768 | 7.26 | 25.14 | 0.794 | 6.73 | 25.77 | 0.789 |
| 10 | 7.68 | 25.32 | 0.758 | 7.24 | 24.60 | 0.782 | 6.68 | 26.28 | 0.811 |
| Mean | 7.65 | 25.62 | 0.763 | 7.27 | 24.70 | 0.785 | 6.67 | 26.41 | 0.803 |

**The data in Table 5**

|  | Loss | PSNR (dB) | SSIM |
| --- | --- | --- | --- |
| MSRAFFB-Net | 5.8 | 33.56 | 0.936 |
| ISR-IR | 6.63 | 31.79 | 0.883 |
| MFFN | 6.73 | 30.3 | 0.838 |
| SRDiff | 6.97 | 31.26 | 0.848 |
| EG-CNN | 6.78 | 31.42 | 0.875 |
| GAN-MSRB | 7.12 | 30.44 | 0.839 |

**The data in Table 6**

| Noise level (σ) | Algorithms | Inference time (ms) | PSNR (dB) | SSIM |
| --- | --- | --- | --- | --- |
| 15 | ISR-IR | 120 | 31.2 | 0.883 |
|  | MFFN | 95 | 30.5 | 0.867 |
|  | SRDiff | 210 | 31.8 | 0.891 |
|  | EG-CNN | 85 | 31 | 0.875 |
|  | GAN-MSRB | 180 | 30.9 | 0.882 |
|  | MSRAFFB-Net | 145 | 33.5 | 0.928 |
| 25 | ISR-IR | 125 | 29.8 | 0.842 |
|  | MFFN | 98 | 28.7 | 0.823 |
|  | SRDiff | 220 | 30.4 | 0.852 |
|  | EG-CNN | 88 | 29.5 | 0.835 |
|  | GAN-MSRB | 190 | 29.3 | 0.841 |
|  | MSRAFFB-Net | 150 | 32.1 | 0.896 |
| 35 | ISR-IR | 130 | 28.1 | 0.801 |
|  | MFFN | 102 | 26.9 | 0.774 |
|  | SRDiff | 235 | 28.7 | 0.812 |
|  | EG-CNN | 92 | 27.6 | 0.789 |
|  | GAN-MSRB | 205 | 27.2 | 0.798 |
|  | MSRAFFB-Net | 160 | 30.6 | 0.863 |

**The data in Figure 8 (a)**

|  | M1 | M2 | M3 | M4 | MSRAFFB-Net |
| --- | --- | --- | --- | --- | --- |
| 0 | 7.41 | 6.64 | 6.87 | 6.79 | 5.84 |
| 1 | 7.31 | 6.68 | 6.82 | 6.75 | 5.75 |
| 2 | 7.29 | 6.72 | 6.93 | 6.71 | 5.85 |
| 3 | 7.34 | 6.70 | 6.97 | 6.68 | 5.83 |
| 4 | 7.39 | 6.74 | 6.95 | 6.67 | 5.75 |
| 5 | 7.30 | 6.74 | 6.84 | 6.76 | 5.77 |
| 6 | 7.37 | 6.63 | 6.86 | 6.75 | 5.82 |
| 7 | 7.40 | 6.71 | 6.86 | 6.76 | 5.84 |
| 8 | 7.42 | 6.64 | 6.89 | 6.73 | 5.75 |
| 9 | 7.35 | 6.64 | 6.97 | 6.72 | 5.85 |
| 10 | 7.40 | 6.66 | 6.84 | 6.78 | 5.74 |

**The data in Figure 8 (b)**

|  | M1 | M2 | M3 | M4 | MSRAFFB-Net |
| --- | --- | --- | --- | --- | --- |
| 0 | 31.30 | 30.04 | 30.91 | 30.95 | 34.01 |
| 1 | 31.68 | 29.88 | 31.21 | 30.49 | 33.81 |
| 2 | 30.97 | 29.56 | 31.10 | 31.47 | 33.12 |
| 3 | 31.70 | 29.97 | 30.57 | 31.79 | 33.37 |
| 4 | 31.10 | 30.46 | 31.57 | 31.29 | 32.94 |
| 5 | 30.78 | 29.58 | 30.44 | 31.63 | 33.33 |
| 6 | 30.38 | 29.97 | 30.30 | 30.70 | 34.38 |
| 7 | 31.42 | 29.87 | 31.01 | 30.55 | 33.27 |
| 8 | 31.01 | 29.81 | 30.64 | 30.45 | 33.78 |
| 9 | 30.99 | 29.83 | 31.67 | 31.73 | 33.48 |
| 10 | 31.13 | 30.88 | 30.59 | 30.53 | 33.72 |

**The data in Figure 8 (c**)

|  | M1 | M2 | M3 | M4 | MSRAFFB-Net |
| --- | --- | --- | --- | --- | --- |
| 0 | 0.816 | 0.879 | 0.842 | 0.867 | 0.943 |
| 1 | 0.820 | 0.878 | 0.841 | 0.872 | 0.930 |
| 2 | 0.816 | 0.883 | 0.849 | 0.872 | 0.938 |
| 3 | 0.820 | 0.868 | 0.849 | 0.875 | 0.928 |
| 4 | 0.815 | 0.874 | 0.838 | 0.875 | 0.940 |
| 5 | 0.808 | 0.882 | 0.846 | 0.876 | 0.942 |
| 6 | 0.806 | 0.875 | 0.840 | 0.867 | 0.936 |
| 7 | 0.819 | 0.870 | 0.848 | 0.868 | 0.934 |
| 8 | 0.819 | 0.882 | 0.843 | 0.867 | 0.932 |
| 9 | 0.820 | 0.873 | 0.846 | 0.869 | 0.938 |
| 10 | 0.818 | 0.869 | 0.849 | 0.870 | 0.937 |

**The data in Figure 9 (a**)

|  | M1 | M2 | M3 | M4 | MSRAFFB-Net |
| --- | --- | --- | --- | --- | --- |
| 0 | 7.41 | 6.64 | 6.87 | 6.79 | 6.00 |
| 1 | 7.31 | 6.68 | 6.82 | 6.75 | 5.91 |
| 2 | 7.29 | 6.72 | 6.93 | 6.71 | 6.00 |
| 3 | 7.34 | 6.70 | 6.97 | 6.68 | 5.99 |
| 4 | 7.39 | 6.74 | 6.95 | 6.67 | 5.91 |
| 5 | 7.30 | 6.74 | 6.84 | 6.76 | 5.93 |
| 6 | 7.37 | 6.63 | 6.86 | 6.75 | 5.98 |
| 7 | 7.40 | 6.71 | 6.86 | 6.76 | 6.00 |
| 8 | 7.42 | 6.64 | 6.89 | 6.73 | 5.91 |
| 9 | 7.35 | 6.64 | 6.97 | 6.72 | 6.01 |
| 10 | 7.40 | 6.66 | 6.84 | 6.78 | 5.90 |

**The data in Figure 9 (b**)

|  | M1 | M2 | M3 | M4 | MSRAFFB-Net |
| --- | --- | --- | --- | --- | --- |
| 0 | 30.68 | 29.84 | 31.48 | 30.56 | 32.07 |
| 1 | 31.06 | 29.60 | 31.72 | 31.20 | 32.73 |
| 2 | 30.61 | 29.51 | 31.00 | 30.44 | 33.34 |
| 3 | 31.22 | 29.57 | 31.41 | 30.95 | 32.40 |
| 4 | 31.05 | 29.94 | 30.88 | 31.06 | 33.40 |
| 5 | 30.91 | 30.31 | 30.45 | 30.81 | 33.08 |
| 6 | 31.48 | 30.05 | 31.13 | 31.63 | 32.06 |
| 7 | 31.26 | 30.46 | 31.23 | 30.55 | 33.11 |
| 8 | 31.30 | 29.83 | 30.65 | 31.00 | 32.23 |
| 9 | 31.54 | 30.21 | 30.53 | 30.48 | 32.31 |
| 10 | 31.30 | 29.92 | 31.48 | 31.06 | 33.25 |

**The data in Figure 9 (c**)

|  | M1 | M2 | M3 | M4 | MSRAFFB-Net |
| --- | --- | --- | --- | --- | --- |
| 0 | 0.818 | 0.868 | 0.851 | 0.866 | 0.917 |
| 1 | 0.814 | 0.869 | 0.842 | 0.871 | 0.906 |
| 2 | 0.806 | 0.880 | 0.840 | 0.878 | 0.909 |
| 3 | 0.806 | 0.868 | 0.842 | 0.866 | 0.918 |
| 4 | 0.820 | 0.870 | 0.840 | 0.865 | 0.918 |
| 5 | 0.806 | 0.881 | 0.850 | 0.873 | 0.906 |
| 6 | 0.807 | 0.878 | 0.847 | 0.870 | 0.916 |
| 7 | 0.806 | 0.879 | 0.839 | 0.867 | 0.912 |
| 8 | 0.812 | 0.868 | 0.852 | 0.870 | 0.912 |
| 9 | 0.820 | 0.879 | 0.846 | 0.875 | 0.905 |
| 10 | 0.811 | 0.883 | 0.853 | 0.865 | 0.917 |

**The data in Figure 10**

|  | M1 | M2 | M3 | M4 | MSRAFFB-Net |
| --- | --- | --- | --- | --- | --- |
| 0 | 72.41 | 81.11 | 88.45 | 80.76 | 88.97 |
| 1 | 73.62 | 80.16 | 85.73 | 80.85 | 90.69 |
| 2 | 70.77 | 81.28 | 79.97 | 76.77 | 91.06 |
| 3 | 76.02 | 79.41 | 79.18 | 78.77 | 88.11 |
| 4 | 75.13 | 76.49 | 83.51 | 80.19 | 91.42 |
| 5 | 71.47 | 75.74 | 86.84 | 81.60 | 89.94 |
| 6 | 68.12 | 78.93 | 80.17 | 75.12 | 89.99 |
| 7 | 74.70 | 80.62 | 86.86 | 75.08 | 90.59 |
| 8 | 68.49 | 76.74 | 86.04 | 78.52 | 89.55 |
| 9 | 73.92 | 76.78 | 86.15 | 78.60 | 89.55 |
| 10 | 71.51 | 78.68 | 87.65 | 75.13 | 91.03 |
| 11 | 69.17 | 80.41 | 82.77 | 76.17 | 90.73 |
| 12 | 72.85 | 77.50 | 87.74 | 78.72 | 89.99 |
| 13 | 73.35 | 79.60 | 80.85 | 77.80 | 90.46 |
| 14 | 70.36 | 79.00 | 79.91 | 76.25 | 88.45 |
| 15 | 73.46 | 78.43 | 79.88 | 77.18 | 90.09 |
| 16 | 72.82 | 81.73 | 87.73 | 77.26 | 89.38 |
| 17 | 75.29 | 75.29 | 82.68 | 76.08 | 89.88 |
| 18 | 76.96 | 80.56 | 86.38 | 75.38 | 91.37 |
| 19 | 68.11 | 76.78 | 83.09 | 79.77 | 89.44 |
| 20 | 76.17 | 80.72 | 84.05 | 76.52 | 88.47 |
| Mean | 72.60 | 78.86 | 84.08 | 77.74 | 89.96 |

The data in Figure 11

|  | M1 | M2 | M3 | M4 | MSRAFFB-Net |
| --- | --- | --- | --- | --- | --- |
| 0 | 64.18 | 69.76 | 78.87 | 70.41 | 85.96 |
| 1 | 65.64 | 67.17 | 75.26 | 69.29 | 85.40 |
| 2 | 60.64 | 66.71 | 80.72 | 75.75 | 85.54 |
| 3 | 59.94 | 69.92 | 76.63 | 72.57 | 83.87 |
| 4 | 62.66 | 68.40 | 78.85 | 68.51 | 85.14 |
| 5 | 64.48 | 74.29 | 75.31 | 73.57 | 83.38 |
| 6 | 67.50 | 67.19 | 77.75 | 74.81 | 83.95 |
| 7 | 64.53 | 74.75 | 75.01 | 71.64 | 83.91 |
| 8 | 63.47 | 71.13 | 81.75 | 70.23 | 85.93 |
| 9 | 67.62 | 73.23 | 75.87 | 70.08 | 83.65 |
| 10 | 59.99 | 67.94 | 75.09 | 74.42 | 85.87 |
| 11 | 63.48 | 66.85 | 81.82 | 66.38 | 83.36 |
| 12 | 58.83 | 69.46 | 80.79 | 72.04 | 84.75 |
| 13 | 67.84 | 71.24 | 76.89 | 72.93 | 83.95 |
| 14 | 59.09 | 70.47 | 81.06 | 75.50 | 83.28 |
| 15 | 64.62 | 68.75 | 80.50 | 74.56 | 85.83 |
| 16 | 67.15 | 68.85 | 80.41 | 71.84 | 83.50 |
| 17 | 62.18 | 67.75 | 80.16 | 74.44 | 85.16 |
| 18 | 58.66 | 69.81 | 75.45 | 70.27 | 85.35 |
| 19 | 65.33 | 75.38 | 75.55 | 74.07 | 85.27 |
| 20 | 59.31 | 69.78 | 75.08 | 66.54 | 84.50 |
| Mean | 63.20 | 69.94 | 78.04 | 71.90 | 84.65 |

**The data in Figure 13**

|  | plant | | | landscape | | |
| --- | --- | --- | --- | --- | --- | --- |
|  | M1 | M2 | MSRAFFB-Net | M1 | M2 | MSRAFFB-Net |
| 0 | 55.73 | 63.37 | 79.12 | 62.25 | 64.84 | 81.35 |
| 1 | 55.89 | 63.51 | 79.19 | 62.55 | 67.69 | 82.59 |
| 2 | 56.14 | 63.81 | 79.21 | 62.59 | 67.72 | 82.61 |
| 3 | 56.34 | 63.83 | 79.33 | 62.68 | 67.82 | 82.71 |
| 4 | 56.94 | 64.36 | 79.47 | 63.37 | 68.36 | 82.79 |
| 5 | 56.99 | 64.54 | 79.68 | 63.67 | 69.00 | 82.84 |
| 6 | 57.61 | 64.98 | 79.71 | 65.14 | 69.10 | 82.97 |
| 7 | 59.07 | 65.31 | 79.75 | 65.48 | 71.03 | 83.26 |
| 8 | 59.53 | 65.41 | 79.75 | 65.59 | 72.06 | 83.29 |
| 9 | 60.30 | 65.99 | 80.28 | 65.80 | 72.51 | 83.35 |
| 10 | 60.31 | 66.27 | 80.51 | 67.67 | 73.12 | 83.38 |
| Mean | 57.71 | 64.67 | 79.64 | 64.25 | 69.39 | 82.83 |
